# Impact of 1q vulnerabilities in patients treated with anti-CD38 monoclonal antibodies

**DOI:** 10.3389/fonc.2026.1758533

**Published:** 2026-03-03

**Authors:** María Sánchez-Tabernero, Irene Zamanillo, José María Sánchez-Pina, Rafael Alonso-Fernandez, Nieves López-Muñoz, Magdalena Corona, Manuela Fernández-Guijarro, María-Teresa Cedena, Susana Cortijo, María Calbacho, Joaquín Martinez-Lopez

**Affiliations:** 1 Department of Hematology, Hospital Universitario 12 de Octubre, Instituto de Investigación Sanitaria Hospital 12 de Octubre (imas12), Madrid, Spain; 2 Clinical Research Hematology Unit, H12O Centro Nacional de Investigaciones Oncológicas (CNIO), Centro de Investigación Biomédica en Red Cáncer (CIBERONC), Madrid, Spain; 3 Faculty Of Medicine, Universidad Complutense de Madrid, Madrid, Spain; 4 Genetic Department, Hospital Universitario 12 de Octubre, Instituto de Investigación Sanitaria Hospital 12 de Octubre (imas12), Madrid, Spain; 5 Pharmacology Department, Hospital Universitario 12 de Octubre, Instituto de Investigación Sanitaria Hospital 12 de Octubre (imas12), Madrid, Spain

**Keywords:** 1q, CD38, daratumumab, isatuximab, multiple myeloma, immunotherapy

## Abstract

Multiple myeloma (MM) is a genetically heterogeneous malignancy in which cytogenetic abnormalities, including gain or amplification of chromosome 1q (+1q), have been associated with adverse outcomes. We conducted a retrospective single-center study evaluating the impact of 1q alterations in MM patients treated with anti-CD38 monoclonal antibodies (daratumumab or isatuximab) between 2015 and 2024. Fluorescence *in situ* hybridization was performed to detect 1q gain or amplification and other high-risk cytogenetic abnormalities. A total of 149 patients were analyzed, with 115 receiving daratumumab and 34 isatuximab. In the daratumumab cohort, 51.3% harbored 1q alterations, with comparable baseline characteristics across groups. Patients with 1q alterations showed a lower rate of MRD negativity (20.3% vs 39.3%; p = 0.04) and a trend toward shorter PFS (26.3 vs 43.4 months; p = 0.05), while OS was similar overall (61.2 vs 68.7 months; p = 0.24), although 1q amplification was associated with poorer OS (42 vs 74 months; p = 0.029). These findings were not confirmed in multivariate analysis, where the line of anti-CD38 therapy emerged as the main variable significantly associated with both PFS and OS. In the isatuximab-treated group, 41% exhibited 1q alterations, with no significant differences in response rates, MRD negativity (28.6% vs 55%; p = 0.171), PFS (56.9 vs 58.2 months; p = 0.67), or OS (median not reached in either group; p = 0.27). The small sample size and low number of events precluded meaningful multivariate analysis. Overall, our findings suggest that 1q alterations are not independent prognostic factors in MM patients treated with anti-CD38 antibodies. The poorer outcomes observed in univariate analyses among daratumumab-treated patients with 1q alterations likely reflect a more advanced disease profile, including later lines of therapy and greater underlying disease risk, rather than the isolated effect of 1q alterations themselves. Comprehensive cytogenetic profiling and achieving MRD negativity remain critical for risk stratification and optimizing therapeutic strategies in MM patients receiving anti-CD38 therapies.

## Introduction

Over the past decades, therapeutic advances have significantly improved survival outcomes in patients with Multiple Myeloma (MM); however, the high biological heterogeneity and genetic complexity remain major challenges in disease management (1, 2). In this context, genetic alterations have gained relevance due to both their high prevalence and prognostic impact.

Among genetic alterations, gain or amplification of the long arm of chromosome 1 (+1q), is one of the most frequent and has consistently been associated with inferior outcomes. The presence of +1q is defined by the acquisition of additional copies of genetic material on chromosome 1q by malignant plasma cells and may occur as whole-arm trisomy or by partial duplications, mostly including the 1q21 region (3). It is important to distinguish between “gain 1q”, defined as one additional copy (three total copies), and “amplification 1q, “ defined as two or more additional copies (four or more total copies) (4). The prevalence of +1q increases across the MM spectrum: it is reported in approximately 17-23% of patients with monoclonal gammopathy of undetermined significance (MGUS), 28-49% of newly diagnosed multiple myeloma (NDMM) patients, and up to 42-80% of relapsed/refractory multiple myeloma (RRMM) cases (4, 5). Studies have shown that the acquisition of +1q is a driver event in MM pathogenesis, but it is also the most common secondary cytogenomic abnormality described during disease progression. This could be explained by the presence of several notable genes located at 1q21, the most frequently implicated region, such as CKS1B, PSMD4, BCL9 and MCL1 (3). Numerous studies have associated +1q with the activation of various genes involved in MM development and cell proliferation. Some of these genes are associated with immune evasion mechanisms, such as the regulation of the complement system. Specifically, overexpression of complement-inhibitory proteins and reduced CD38 expression on the surface of myeloma cells, often driven by 1q alterations, have been associated with resistance to anti-CD38 monoclonal antibodies (4). Furthermore, the extent of 1q gain or amplification and the clonal proportion of cells carrying this alteration correlate with greater disease aggressiveness and suboptimal response to conventional therapies (4, 6).

The current prognostic model for MM incorporates several cytogenetic markers, including deletion of chromosome 17p and the t(4;14) and t(14;16) translocations, which have traditionally been considered high-risk. Recently, 1q gain/amplification has been recognized as another significant adverse factor. In this regard, +1q has emerged as a risk marker, as its presence is associated with shorter progression-free survival (PFS) and, in many cases, reduced overall survival (OS) (7). Its inclusion in staging systems such as the second revision of the International Staging System (R2−ISS) underscores the importance of this alteration for risk stratification and therapeutic decision-making (8). However, the prognostic relevance of +1q appears to be modulated by the presence of co-occurring high-risk features rather than acting as an isolated determinant of outcome (9).

In the current therapeutic landscape, anti-CD38 monoclonal antibodies have become a cornerstone of modern MM therapy. Currently, two anti-CD38 monoclonal antibodies, daratumumab, a fully human IgG1 monoclonal antibody, and isatuximab, a humanized chimeric anti-CD38, are approved and widely used in clinical practice.

Direct comparative evidence between daratumumab and isatuximab in patients with 1q alterations is limited. Nevertheless, some studies have suggested that isatuximab may be more effective in this subgroup, possibly due to its mechanism of action, which relies on antibody-dependent cytotoxicity and other immunomodulatory mechanisms, potentially resulting in a more consistent response in patients with 1q abnormalities. By contrast, daratumumab exerts its activity primarily via complement-dependent cytotoxicity, and for this reason it has been suggested that it may be less effective in this patient subgroup (4).

In retrospective studies and *post hoc* analyses, the addition of daratumumab to standard regimens has been shown to improve PFS in patients with +1q. However, the magnitude of this benefit may be attenuated in cases involving additional 1q copies or other high-risk cytogenetic abnormalities (10). For instance, some analyses have shown that while patients with gain 1q demonstrate improvement, those with amplification may experience a less favorable response, suggesting that the extent of the alteration has both prognostic and therapeutic implications (3). On the other hand, preliminary data with isatuximab have shown promising results in terms of response and survival in the RRMM setting, even in subgroups with +1q, raising the hypothesis that its mechanism of action may counteract, at least partially, the adverse effects of this alteration (4).

Our aim was to evaluate the impact of 1q gain or amplification in multiple myeloma patients treated with anti-CD38 monoclonal antibodies in a real-life cohort of patients treated in a third level center.

## Methods

### Study design

We conducted a retrospective cohort study at a single center, including multiple myeloma patients who received daratumumab or isatuximab as part of any treatment regimen between 2015 and 2024.

Fluorescence *in-situ* hybridization (FISH) was performed on bone marrow samples from all patients, using the last available sample prior to initiation of anti-CD38 therapy, to detect 1q21 gain or amplification in accordance with the International Myeloma Working Group (IMWG) criteria. Other high-risk cytogenetic alterations were also assessed, including t(4;14), t(14;16), t(11;14), and deletion of chromosome 17p and 1p along with additional clinically relevant variants. CD138+ plasma cells were isolated from these samples using Miltenyi Biotec AutoMACS Pro Cell Separator and FISH analyses were subsequently carried out on the isolated cells using the ThermoBrite^®^ Abbott Molecular system. A cutoff of 10% was applied for all cytogenetic alterations, following the recommendations of Grupo Español de Mieloma (GEM).

Additional clinical and laboratory variables were collected prior to initiation of anti-CD38 therapy, including serum albumin, lactate dehydrogenase (LDH), and β2-microglobulin levels.

### End point definitions

The effectiveness endpoints assessed were progression-free survival (PFS), overall survival (OS), progression rate and response rates (complete response [CR] and minimal residual disease [MRD]). Responses to therapy were assessed using the International Myeloma Working Group criteria (11). Progression rate was defined as a binary outcome (yes/no), corresponding to the occurrence of disease progression at any time during the follow-up period, irrespective of timing. PFS was defined as the interval from the start of treatment to documented disease progression or death from any cause, whichever occurred earlier. OS was defined as the time from treatment initiation to death from any cause, or to the date of last follow-up for patients who were still alive.

### Statistical analyses

Baseline characteristics were compared separately within the isatuximab and daratumumab groups using chi-square tests. These comparisons were descriptive and not intended to assess treatment efficacy.

Associations between 1q alteration status (presence vs absence) and categorical clinical outcomes, including complete response (CR), minimal residual disease (MRD) status and progression rate, were also assessed using chi-square tests. These analyses were descriptive in nature and were not intended to compare treatment efficacy between isatuximab and daratumumab or to estimate effect sizes.

Time-to-event outcomes (PFS and OS) were analyzed using the Kaplan–Meier method and compared with the log-rank test.

Multivariable Cox proportional hazards regression models were constructed for PFS and OS using baseline variables only, including 1q alteration status, the presence of additional high-risk cytogenetic abnormalities included in ISS-R (12) (defined as ≥1 of del(17p), t(14;16) or t(4;14)), extramedullary disease, and line of anti-CD38 therapy. Line of therapy was included as a categorical variable (first line as reference). These analyses were conducted as exploratory to identify independent baseline prognostic factors.

Multivariable Cox proportional hazards regression analyses were not performed in the isatuximab-treated cohort due to the small sample size and limited number of events, which would not allow reliable estimation of multivariable models.

Exploratory Kaplan–Meier analyses according to MRD status were also performed within each treatment cohort.

All statistical analyses were performed using SPSS^®^ software.

### Ethical consideration

This study was conducted following the Declaration of Helsinki and the International Conference on Harmonization (ICH) Guidelines for Good Clinical Practice and was approved by our ethics committee (No. 20/326).

## Results

### Patient characteristics

We analyzed a total of 149 patients, of whom 115 were treated with daratumumab and 34 with isatuximab (**Table 1**).

**Table 1:** Baseline characteristics of multiple myeloma patients.

	Daratumumab, N = 115	Isatuximab, N = 34
Gender		
Women, n (%)	63 (54.8)	20 (58)
Men, n (%)	52 (45.2)	14 (42)
Age at diagnosis, median (range)	66 (18-90)	61 (38-78)
ISS-R at diagnosis, n (%)		
Stage I	28 (24.3)	11 (32.4)
Stage II	39 (33.9)	14 (41.2)
Stage III	27 (23.5)	5 (14.7)
Unknown	21 (18.3)	4 (11.8)
Extramedullary disease	19 (16.5)	6 (17.6)
Cytogenetic profile at diagnosis, n (%)		
Del17p	20 (17.4)	7 (20.5)
t (4;14)	13 (11.3)	8 (23.5)
t (14;16)	5 (4.3)	0 (0)
t (11;14)	14 (12.2)	0 (0)
Del1p	12 (10.4)	3 (8.8)
Monosomy 13	20 (17.4)	4 (13.6)
1q+, n (%)	59 (51.3)	14 (41)
Gain	31 (27)	7 (20.5)
Amplification	28 (24.3)	7 (20.5)
Line of therapy, median (range)	3 (1-8)	2 (1-7)
First-line, n (%)	36 (31.3)	16 (47)
Second-line, n (%)	36 (31.3)	11 (32.3)
Third-line, n (%)	24 (20.9)	4 (11.8)
Fourth-line n (%)	15 (13)	>4 (8.7)
Previous proteasome inhibitor, n (%)	72 (62.6)	18 (53)
Previous immunomodulatory drug, n (%)	68 (59.1)	18 (53)
Previous ASCT, n (%)	42 (36.8)	11 (32)
Treatment regimen		
	DKd 21 (18.3)	IKd 14 (41.2)
	DVd 20 (17.4)	IKRd 5 (14.7)
	DVMP 18 (15.7)	IVRd 5 (14.7)
	DRd 14 (12.2)	IVCd 4 (11.8)
	Dd 14 (12.2)	
	DPd 11 (9.6)	
Clinical trial, n (%)	47 (38)	19 (55.8)

In the daratumumab-treated cohort, 51.3% had 1q alterations (27% of the patients exhibited 1q gain and 24.3% had 1q amplification). Other additional cytogenetic abnormalities evaluated were present at frequencies comparable to those reported in large cohorts of multiple myeloma; 17.4% of patients had del17p, 11.3% harbored t(4;14), 12.2% had t(11;14), 10.4% exhibited del(1p) and 4.3% had t(14;16). A considerable proportion of patients exhibited high risk characteristics prior to anti-CD38 therapy, such as ISS-R stage 3 in 23.5% of these patients, and extramedullary disease in 16.5% of the cohort. Baseline characteristics were generally comparable between patients with and without 1q alterations, although the former group more frequently showed ISS-R stage 3 (not statistically significant), del(17p) (not statistically significant), and t(4;14) (p = 0.05).

Regarding treatment regimens, daratumumab was predominantly administered in combination with either carfilzomib (18.3%), or bortezomib (36.1%). Daratumumab in monotherapy was given to 12.2% patients, and no patient received a quadruplet regimen. The median of line of treatment was 3 (IQR 1-8), 62.6% of patients had been previously treated with proteasome inhibitors, 59.1% with IMiDs (immunomodulatory drugs), and 36.8% had undergone ASCT (autologous stem cell transplant). Only 31.3% of patients received daratumumab as a first-line treatment.

In the isatuximab-treated group, 1q alterations were present in 41% of the patients (20.5% had 1q gain and 20.5% had 1q amplification). This was a slightly lower risk cohort with ISS-R stage 3 present in 14.5% of the patients and extramedullary disease found in 17.6%.

Baseline characteristics were similar between patients with and without 1q abnormalities in this subgroup, although those harboring 1q alterations were more likely to present with high-risk ISS-R (not statistically significant) and t(4;14) (p = 0.004). Aside from 1q alterations, t(4;14) was the most frequently observed cytogenetic abnormality (23.5%), followed by del(17p) (20.5%) and del(1p) (8.8%). Regarding treatment lines, 47% of patients received isatuximab as first-line therapy, 32.3% as second-line therapy and 11.8% as third-line therapy. The most used combination regimens included isatuximab with carfilzomib (41.2%), with bortezomib and lenalidomide (14.7%) and with bortezomib and cyclophosphamide (11.7%). Also, 29.4% of patients received a quadruplet regimen and no patient received isatuximab in monotherapy. Prior to isatuximab treatment, 53% of patients had received proteasome inhibitors, 53% had been treated with IMiDs and 32% had undergone ASCT.

### Impact of 1q in the efficacy of patients treated with anti-CD38 therapies

Outcome analyses for the daratumumab-treated patients demonstrated no significant differences in progression rates or complete response (CR) rates when comparing patients with 1q alterations (gain or amplification) to those without (71.1% vs 57.1% p = 0.116 for progression rate and 55.1% vs 67.2 p = 0.187 for CR rate). However, patients with any form of 1q alteration exhibited a significantly lower rate of minimal residual disease (MRD) negativity (20.3% vs 39.3%; p = 0.04). Analyzing separately patients with 1q gain and 1q amplification, no statistically significant differences were observed regarding CR or progression rates. A trend was observed regarding the rate of MRD negativity among patients with 1q amplification (14.3% vs 34.5%; p = 0.056).

**Figure 1:**
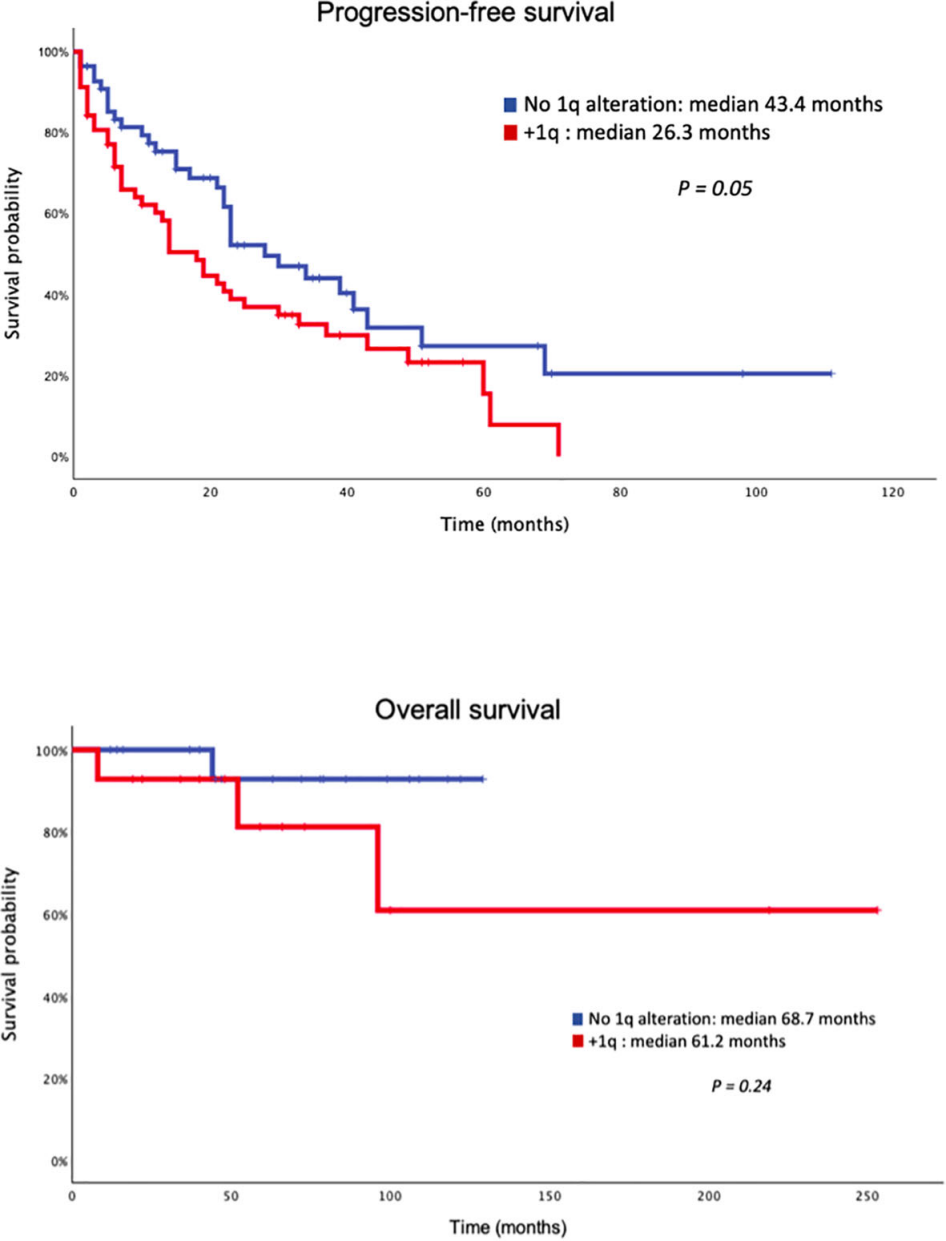
Progression-free survival and overall survival of daratumumab treated patients.

Survival analysis (see **Figure 1**) indicated a trend toward shorter PFS (progression free survival) in patients with 1q alterations (including both gain and amplification) with 26.3 months versus 43.4 months in those without 1q alterations (p = 0.05). Regarding overall survival, no significant differences were found (61.2 months vs 68.7 months; p = 0.24). Furthermore, patients with 1q amplification presented worse outcomes, with a median OS of 42 months compared to 74 months for patients without 1q amplification (p = 0.029).

MRD negativity post treatment was strongly associated with improved outcomes. Median PFS was 51.0 months in MRD-negative patients compared with 14.0 months in MRD-positive patients (log-rank p < 0.001). MRD negativity was also associated with significantly longer OS; median OS was 43.0 months in MRD-positive patients, whereas median OS was not reached in MRD-negative patients (log-rank p < 0.001).

In the isatuximab-treated group, no statistically significant differences were observed in progression rates, CR rates, or MRD negativity between patients with and without 1q alterations (36.3% vs 17.8% p = 0.140 for progression rate, 54.5% vs 75% p = 0.130 for CR rate and 28.6% vs 55% p = 0.171 for MRD rate). Separate analyses for 1q gain or amplification revealed no differences in CR, MRD or progression rates.

**Figure 2:**
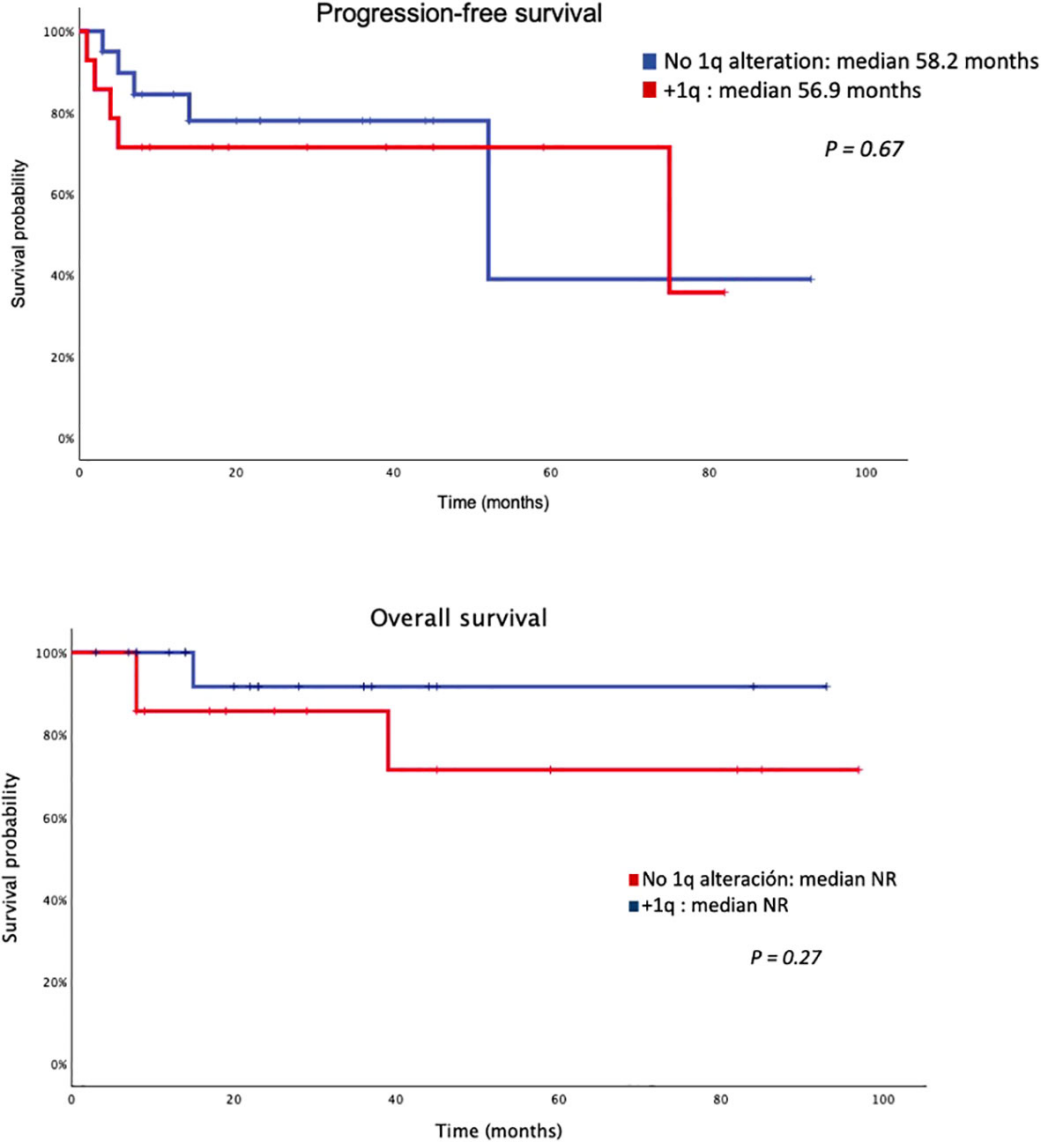
Progression-free survival and overall survival of isatuximab treated patients.

With respect to survival analysis (see **Figure 2**), no significant differences regarding PFS were found in overall 1q alteration status (including 1q gain or 1q amplification): 56.9 months vs 58.2 months (p = 0.67). Nevertheless, a trend was observed in the median PFS for 1q amplification cases (35 months vs 64 months; p = 0.24). Overall survival among isatuximab-treated patients was not significantly affected by 1q status. Median OS was not reached in patients with or without 1q alterations, and no statistically significant differences were observed between groups (log-rank p = 0.27). When analyzing 1q gain and 1q amplification separately, median OS was also not reached in either subgroup, with no significant differences identified.

In the isatuximab-treated cohort, MRD negativity was associated with longer PFS (log-rank p = 0.036); however, this finding should be interpreted with caution, as only one progression event occurred among MRD-negative patients, precluding reliable estimation of median PFS. OS analyses according to MRD status were not informative due to the absence of death events during follow-up.

In multivariate analysis (see **Table 2**), +1q was not confirmed as an independent prognostic factor for PFS or OS in the daratumumab-treated cohort after adjustment for other high-risk cytogenetic abnormalities, extramedullary disease, and line of therapy. Line of treatment emerged as a significant predictor of outcome, with later treatment lines being associated with inferior survival. The presence of additional high-risk cytogenetic abnormalities, rather than 1q status alone, accounted for the differences observed in univariate analyses.

**Table 2:** Multivariate analysis of daratumumab treated patients.

Progression-free survival – Multivariate analysis		
Characteristic	HR (95% CI)	P-value
1q alteration	1.39 (0.86 – 2.23)	0.180
Additional high-risk cytogenetic abnormalities	0.99 (0.59 – 1.66)	0.972
Extramedullary disease	1.69 (0.90 – 3.17)	0.104
Line of therapy: 2L vs 1L	1.34 (0.71 – 2.54)	0.368
Line of therapy: ≥3L vs 1L	**2.18 (1.24** – **3.84)**	**0.007**
Overall survival – Multivariate analysis		
Characteristic	HR (95% CI)	P-value
1q alteration	1.21 (0.72 – 2.06)	0.473
Additional high-risk cytogenetic abnormalities	1.10 (0.62 – 1.95)	0.744
Extramedullary disease	1.88 (0.99 – 3.57)	0.054
Line of therapy: 2L vs 1L	**2.63 (1.20** – **5.76)**	**0.016**
Line of therapy: ≥3L vs 1L	**2.82 (1.35** – **5.87)**	**0.006**

Multivariable Cox regression models were adjusted for baseline variables including 1q alteration (gain or amplification), additional high-risk cytogenetic abnormalities (≥1 of del(17p), t(14;16), t(4;14)), extramedullary disease, and line of daratumumab therapy. Line of therapy was included as a categorical variable (1L as reference). Only baseline variables were included in the multivariable models.

Bold values indicate variables that remained statistically significant in multivariate analysis.

As explained in methods, the small number of patients in the isatuximab subgroup, combined with the low number of events, prevented reliable estimation of a multivariate Cox model. As a result, the findings from this analysis could not be interpreted meaningfully.

## Discussion

In our study, in the daratumumab-treated cohort, patients with 1q gain or amplification showed a trend towards a shorter PFS and patients with 1q amplification presented with a shorter OS, consistent with the concept that amplification reflects a more advanced cytogenetic event. These differences were not evident in the isatuximab-treated group, suggesting that the adverse prognostic impact of 1q alterations may be less pronounced in this cohort, a finding consistent with previous reports (13). However, once additional risk markers were considered in the multivariate analysis in daratumumab group, the isolated prognostic impact of 1q alterations was largely abrogated. This finding implies that the inferior outcomes observed in some daratumumab-treated patients are likely due to the cumulative burden of adverse cytogenetic events and other high-risk factors rather than the effect of +1q alone.

In our cohort, patients with 1q gain or amplification, although generally comparable to the general cohort, also presented with other high‐risk features like deletion of 17p, t(4;14), and higher ISS‐R scores in general, though statistically significant differences were noted only for t(4;14), which was present in a very low number of patients (11.3% in daratumumab group and 23.5% in isatuximab group). These additional abnormalities have been associated with shorter PFS and reduced OS in previous studies (5), although when evaluated together in our multivariate analysis, none reached statistical significance as independent factors.

Moreover, as outlined in the introduction, the extent of 1q gain or amplification and the clonal proportion of cells carrying this alteration may correlate with greater disease aggressiveness and suboptimal response to conventional therapies (4, 6). This suggests that patients with 1q amplification, rather than simple gain, may have a worse prognosis, reflecting a more advanced acquired cytogenetic alteration. This trend was observed in the univariate analysis of our daratumumab cohort, where patients with 1q amplification had poorer overall survival, a pattern not seen in patients with 1q gain alone. Additionally, MRD negativity was less frequently achieved in patients with 1q amplification, whereas in those with 1q gain, response rates were like the overall cohort.

Importantly, in an exploratory analysis, MRD negativity emerged as a robust prognostic marker, showing a strong association with both PFS and OS in daratumumab-treated patients. These findings support the role of MRD as a powerful indicator of treatment benefit in MM (14).

In daratumumab-treated patients, multivariate analysis showed that 1q status (including both gain and amplification) was not confirmed as an independent prognostic factor. In contrast, the line of treatment in which patients received daratumumab had a strong and significant impact on both PFS and OS. Additionally, the presence of extramedullary disease demonstrated a trend toward shorter overall survival, although this did not reach statistical significance. These results suggest that the differences observed in the univariate analysis (including a trend toward shorter PFS in +1q patients) may reflect a more advanced disease state with a higher-risk profile, including the presence of greater cumulative cytogenetic burden and later line of treatment.

This observation highlights the importance of evaluating the complete cytogenetic profile rather than focusing on a single alteration. It is plausible that the overall genetic complexity, rather than the presence of a solitary 1q gain or amplification, underlies the reduced efficacy of daratumumab in certain subgroups of MM. In support of this notion, other studies have demonstrated that the intensity of the adverse prognostic impact increases with the number of high-risk alterations present, suggesting a cumulative effect rather than a singular driver (3, 8).

In summary, it has been suggested that the high risk associated with +1q could be mitigated with the use of isatuximab instead of daratumumab. However, our analysis suggests that the less favorable outcomes observed among patients receiving daratumumab are not driven by 1q alterations alone, but by the cumulative burden of additional high-risk cytogenetic abnormalities.

While our study contributes to the growing body of evidence that the adverse impact on outcomes in daratumumab-treated MM patients is more complex than the presence of +1q alone, further research is warranted. Prospective randomized trials that include detailed cytogenetic analyses will be pivotal to validate these findings.

Emerging technologies such as next-generation sequencing and single-cell analysis may offer deeper insights into the clonal evolution of MM and help refine our understanding of how multiple high-risk events interact to influence therapeutic efficacy.

The main limitations of this study include its retrospective design, the heterogeneous patient population, and the relatively small sample size, which may limit the generalizability of the findings and the statistical power to detect certain associations.

In summary, while +1q alterations are associated with adverse outcomes in MM, our findings strongly suggest that the less favorable outcomes observed in some daratumumab-treated patients are not driven solely by the presence of 1q gain or amplification, but rather by a higher-risk profile, including greater cumulative cytogenetic burden and later line of treatment. Consequently, comprehensive cytogenetic evaluation is essential for risk stratification and therapy selection, and future studies should focus on stratifying patients based on the entire spectrum of high-risk genetic alterations to optimize the use of anti-CD38 monoclonal antibodies in MM and other novel therapies.

## Data Availability

The raw data supporting the conclusions of this article will be made available by the authors, without undue reservation.
